# Adverse drug reactions of nonsteroidal anti-inflammatory drugs in orthopedic patients

**DOI:** 10.4103/0976-500X.77104

**Published:** 2011

**Authors:** Alpa Pragnesh Gor, Miti Saksena

**Affiliations:** *Department of Pharmacology, Pramukh Swami Medical College, Gokalnagar, Karamsad 388 325, Anand, Gujarat, India*

**Keywords:** Adverse drug reaction, pharmacovigilance, nonsteroidal anti-inflammatory drug

## Abstract

**Objectives:**

To identify the ADRs due to NSAIDs and to know how to monitor the drug’s effect.

**Materials and Methods:**

A descriptive study was undertaken in the Orthopedic Outpatients Department of a tertiary care teaching hospital. Hundred patients were enrolled in this study to observe the risk of adverse drug reactions (ADRs) due to NSAIDs. All the ADRs were further analyzed in relation to age and sex, type of drug and its pattern. Probability scale was used for the causality assessment of the ADRs.

**Results:**

26% of the 100 patients developed ADR due to NSAIDs. There was not much of a difference in the number of the ADRs in relation to the gender. Diclofenac was the highest prescribed drug (65 patients), followed by paracetamol (12), nimesulide (10), ibuprofen (6), piroxicam (5) and Etoricoxib (2). Diclofenac accounted for the maximum number (73%) of ADRs, followed by nimesulide (16%), paracetamol (7%), and Etoricoxib (4%).

**Conclusion:**

Pharmacovigilance improves recognition of ADRs by the medical students. It allows the treating physician to identify the ADR associated with drugs, in particular, with the ones considered relatively safe and with those commonly prescribed by the medical and non-health professionals.

## INTRODUCTION

Nonsteroidal anti-inflammatory drugs (NSAIDs) have been used to decrease pain and inflammation for rheumatological and other conditions for decades. They cause side effects including gastrointestinal (GI) disorders (from minor dyspepsia through to major ulcers, bleeding and perforation), kidney effects and cardiovascular effects. NSAIDs are a group of drugs that inhibit both the isoforms of cyclooxygenase enzyme (COX-1 and COX-2). Conventional NSAIDs are nonselective, which bind and inhibit both the isoforms, but cyclooxygenase-1 (COX-1) is inhibited more avidly than cyclooxygenase-2 (COX-2).[1] Inhibition of COX-1 is responsible for the side effects and that of COX-2 for therapeutic effects. This has resulted in the introduction of the COX-2 selective drugs.[2]

For patients with musculoskeletal disorders, conventional NSAIDs form the mainstay of clinical care.[3]

Some risks of traditional NSAIDs were well documented before the development of selective COX-2 inhibitors. NSAID induced gastric perforations, obstructions and ulcers were blamed for the 16.000 deaths that occurred in the US and 10,000 deaths in Canada in a year.[4]

It has been proposed that COX-2 inhibitors result in anti-inflammatory and analgesic properties, similar to what can be achieved with conventional NSAIDs. However, by sparing COX-1 activity, selective COX-2 inhibitors have greatly reduced toxicity, particularly in GIT. The prevalence of NSAID induced ulcer has been reported to be between 10 and 25% and it causes significant morbidity and mortality.[5]

The pattern of adverse drug reactions (ADRs) caused by NSAIDs in different organ systems is essentially similar. The main distinctions are in the quantitative differences that exist in the occurrence or frequency of ADRs among the different groups, especially those more frequently occurring in the GI tract, liver and, to some extent, kidney.[6–12] Some drugs do have a propensity to cause rare ADRs, e.g., agranulocytosis and aplastic anaemia with phenylbutazone,[6,7] and Stevens Johnson and Lyell’s Syndromes and other severe skin reactions with isoxicam and piroxicam.[13,14] The difficulty is to quantify many of the individual reactions, especially when it comes to population studies.[7] Here, the main issue is to establish the exposure of a known population to an individual drug and to know if individual members of population are taking other drugs or have conditions that might contribute to, or be major confounding factors, in the development of ADRs.[6,7,13,15]

In the backdrop of these issues, the study was undertaken to identify the ADRs due to NSAIDS and to know how to monitor the drug’s effect.

## MATERIALS AND METHODS

The descriptive study was conducted for monitoring the ADRs in the Orthopedic Outpatients Department of Shree Krishna Hospital and Medical Research Centre, a 550-bedded tertiary care teaching rural hospital attached to Pramukhswami Medical College, Karamsad, after obtaining the approval of the Institutional Ethics Committee. The study period was of 3 months duration, from June 2009 to 31 August 2009. The effective sample size was 97 after assuming incidence rate of ADR, which was 10%.[16] In our study, the sample size was 100 patients.

The concerned specialists of the orthopedic department were informed about the aim of the study, seeking their cooperation and were assured of full confidentiality of the information. A total of 100 patients taking NSAIDs, of either sex, of age 18 years and above were included in the study. The following categories of patients were excluded from the study: patients with history of liver and kidney damage, cardiovascular disease, acid peptic diseases, pregnancy and lactation. All the patients were enrolled after getting their informed and written consent as per the inclusion and exclusion criteria. Demographic data, medical history and diagnosis were noted. Detailed history of ADR (drug name, dose and frequency, date of onset, pattern) was recorded in separate performa. Naranjo probability scale was used for the causality assessment of the ADRs. No follow-up was done. Data were analyzed using Fischer’s exact test. A *P* value <0.05 was consider significant.

## RESULTS

Out of 100 patients, 26 had developed ADR. There was not much difference in the number of patients in relation to gender. When the number of males and females was compared, there was no significant difference between the two groups (*P* > 0.001) [Table 1]. The number of patients who received diclofenac was more in comparison to the number that received other NSAIDs. As the number of prescriptions of diclofenac was more, the percentage of ADR was more [Table 2]. Out of 26 ADRs, 19 (73%) were due to diclofenac, 2 (7%) were due to paracetamol and 4 (15%) were due to nimesulide [Table 2]. Less number of patients received ibuprofen or piroxicam, and therefore, no ADR was found due to these drugs. Out of two patients, one had ADR due to etoricoxib. Nineteen ADRs were found in the age group between 18 and 65 years and seven were in the age group above 65 years, which was found to be statistically significant (*P* < 0.001) [Table 3]. When the two groups were compared in relation to gender and occurrence of ADR, it was found to be statistically insignificant [Table 4]. This implies that gender has no effect on the occurrence of ADRs due to NSAIDs. Out of 26 ADRs, 9 were “Probable,” 12 were “Possible” and 5 were “Doubtful’ in nature after causality assessment.

**Table 1:** Age and sex of the patients

Age group	Males *n* (%)	Females *n* (%)	Total *n*
I (18–65 years)	54 (60)	35 (40)	89
II (>65 years)	5 (45.5)	6 (54.5)	11
Total	59	41	100

χ^2^: 9.008, *P* value >0.003

**Table 2:** Drug utilization and adverse drug reaction

Name of drugs	Number of patients	Number of ADRs n (%)
Diclofenac	65	19 (73)
Paracetamol	12	2 (7)
Nimesulide	10	4 (16)
Ibuprofen	6	0 (0)
Etoricoxib	2	1 (4)
Piroxicam	5	0 (0)
Total	100	26 (100)

ADR-adverse drug reactions

**Table 3:** Age of patients and adverse drug reactions

Age group (years)	Number of patients		
	With ADR n (%)	Without ADR n (%)	Total
18–65	19 (22)	70 (78)	89
>65	7 (64)	4 (36)	11
Total	26	74	100

Fischer’s exact test – relative risk = 0.34 with 95% CI (0.18–0.61) and *P* value as 0.006, ADR-adverse drug reactions

**Table 4:** Sex of patients and adverse drug reactions

	Number of patients			
Sex	With ADR n (%)	Without ADR n (%)	Total
Males	15 (25)	44 (75)	59
Females	11 (26)	30 (74)	41
Total	26	74	100

Fisher’s exact test – relative risk = 0.95 with 95% CI (0.48–1.8) and *P* value as 1.00, ADR-adverse drug reactions

## DISCUSSION

On the basis of the present study, it was found that the prevalence rate of ADR was 26%. A study conducted in Mumbai showed that the incidence rate of various kinds ADRs of NSAIDs was ranging from 28 to 33%.[17] The specific risk factors for NSAID ADR are older age, a history of gastro-duodenal ulcer, dyspepsia, concomitant use of medications such as corticosteroids and anticoagulants, high dosage use of multiple NSAIDs and the presence of other chronic co-morbidities.[18] In our study, only 11 patients were above 65 years of age, who received NSAIDS. Among these 11 patients, 7 (64%) developed ADR. Although one of these risk factors (i.e., age) was statistically significant, further clinical review is required to see if they are applicable to clinical practice. Risk factor models for NSAIDs associated with gastropathy were constructed.[19] These models should be helpful to clinicians in predicting possible GI toxicity when they prescribe NSAIDs. These results prompt us to hypothesize that if more source materials are available and more risk factors are identified, we can establish models to predict the overall occurrences of ADRs induced by NSAIDs, which could be more valuable to clinical practice. We have not evaluated other risk factors. In the present study, it was found that diclofenac, paracetamol, nimesulide, ibuprofen, etorocoxib and piroxicam were the top six orally taken NSAIDs. Diclofenac was the most prescribed drug. One review article suggested that an agent with comparatively less GI side effects, like ibuprofen and diclofenac, should be preferred for indomethacin, piroxicam or naproxen, which are more gastro toxic.[1] In our study, out of 65 patients, 19 developed ADRs due to diclofenac. Out of these 19 patients, 14 had GIT related symptoms (i.e., nausea, abdominal distress, gastritis, vomiting, etc.). Three patients had skin-related symptoms (i.e., urticaria, itching and redness of skin) and two patients developed non-specific symptoms (i.e., burning in body parts and general weakness). No ADR was found in all six patients who received ibuprofen. A review suggests that in situations like osteoarthritis where inflammation of joint is minimal, analgesics like paracetamol should be preferred over anti-inflammatory drugs like ibuprofen.[20] In our study, 12 patients received paracetamol. Out of these, two developed ADR (i.e., decreased appetite and abdominal distress in each). All osteoarthritis patients received paracetamol. Since the number of patients who received diclofenac was more in comparison to the number that received other NSAIDs, the number of ADRs was more with diclofenac. Studies have reported that though 20% of patients on long-term treatment with diclofenac experience side effects, only 2% have to discontinue the drug, mostly due to GI complaints.[21] Nimesulide is the second drug causing the highest ADRs, as it accounts for the same in 4 out of 10 patients. The symptoms of ADR due to nimesulide were nausea, decreased appetite, vertigo and general weakness, which suggested no concluding remark. Though nimesulide is a preferential COX-2 inhibitor, and therefore assumed to be safer in clinical use, its GI tolerance has not been proven to be superior to other NSAIDs because various epidemiological studies give little importance to the hypothesis that selective inhibition of COX-2 may have a sparing effect on the GIT.[22] In our study, Etoricoxib was not adequate to draw any conclusions because of the very low number of prescriptions (only two patients). As such, Etoricoxib does not have direct topical effect on the GI mucosa, which confers less risk of the clinical manifestation of GI toxicity typically seen with nonselective NSAIDs, supporting the concept that sparing the COX-1 results in a lack of interference with platelet functions and gastroprotective mechanisms.[23] Several publications indicate that the female gender experiences a higher incidence of ADRs than does the male gender.[24] In our study, there was not much difference in the number of ADRs in male and female patients. Although the study was conducted on limited number of patients and in a short period of time without follow-up, we could not conclude anything regarding relation of gender and ADR.

## CONCLUSION

Monitoring of ADRs is an ongoing, ceaseless, and continuing process. Though pharmacovigilance is still in its infancy in India, this is likely to expand in the times to come. This is because as the newer and newer drugs hit the market, the need for pharmacovigilance grows more than ever before. Pharmacovigilance is an important tool for the treating physician to develop safe medical practice. Identifying the adverse drug events, recording them meticulously and reporting them to the concerned authority is a valuable task in medical profession. This practice will prove to be very valuable in making the drug therapy safer and rational. This study has paved the way to carry out further studies on a large population in the future.
